# Hahnemann, the Founder of Scientific Therapeutics

**Published:** 1883-04

**Authors:** 


Hahnemann, the Founder of Scientific Therapeutics. By R. E. Dudgeon, M. D. Pp. 112. London: E. Gould & Son. 1882.

Some years ago the project of having an annual lecture delivered before the "London School of Homœopathy" was originated. The first "Hahnemannian Lecture"—as they are called—was delivered by Dr. Burnett; subject, Hahnemann as a Man and Physician. The second, by Dr. Hughes, discussed Hahnemann as a Medical Philosopher. In delivering this, the third lecture, Dr. Dudgeon considers Hahnemann as the Founder of Scientific Therapeutics.

After giving a brief history of medicine in its earliest days and at Hahnemann's time, the lecturer proceeds to narrate Hahnemann's experiments and the law he based on them.

The learned lecturer evidently believes only in a crude kind of Homœopathy—the *rule* similia, the single remedy, and the small dose. He prefers the teaching of the beginner to that of the experienced savant! We quote some sentences to show the evident bias of the book: "Fortified by his (Hahnemann's) nine years of diligent experimentation with medicines, in order to ascertain their pathogenetic powers, and his equally long trials of the curative powers of medicines given on the *similia similibus* principle, he felt himself justified in declaring this therapeutic rule to be of general application, and the use of palliatives to be limited to tiding over temporary difficulties, such as apparent death from freezing or asphyxia and hysterical convulsions" (pp. 27 and 28).

Again, "A consideration of Hahnemann's writings during his period of banishment in Cœthen forces on us the conclusion that his expulsion from Leipzic was an unmitigated misfortune for Homœopathy. It was during this period that he introduced all those surprising doctrines that have rendered his system so repugnant to the intelligence of educated physicians. He then abandoned the safe and sure path of observation and experiment for the hazardous and unprofitable way of speculation and hypothesis, the folly of which he had previously demonstrated. He adopted the hypothesis of diseases being a derangement of the vital force; of the spirit of a medicine being liberated from the matter; of the increase of power of medicine by trituration and succussion; of the origin of chronic diseases from three fixed miasms; of the antipsoric power of certain substances, and of a certain dilution being the appropriate dose for all medicines in all diseases."

These quotations give a fair idea of the views of the lecturer. Comment is unnecessary.

